# Hydrophobic Chitosan Nanoparticles Loaded with Carvacrol against *Pseudomonas aeruginosa* Biofilms

**DOI:** 10.3390/molecules27030699

**Published:** 2022-01-21

**Authors:** Ariadna Thalia Bernal-Mercado, Josué Juarez, Miguel Angel Valdez, Jesus Fernando Ayala-Zavala, Carmen Lizette Del-Toro-Sánchez, David Encinas-Basurto

**Affiliations:** 1 Departamento de Investigación y Posgrado en Alimentos, Universidad de Sonora, Blvd. Luis Encinas y Rosales S/N, Col. Centro, Hermosillo 83000, Mexico; thalia.bernal@unison.mx (A.T.B.-M.); carmen.deltoro@unison.mx (C.L.D.-T.-S.); 2 Departamento de Física, Posgrado de Nanotecnología, Universidad de Sonora, Blvd. Luis Encinas y Rosales S/N, Col. Centro, Hermosillo 83000, Mexico; josue.juarez@unison.mx (J.J.); miguel.valdes@unison.mx (M.A.V.); 3 Coordinación de Tecnología de Alimentos de Origen Vegetal, Centro de Investigación en Alimentación y Desarrollo, A.C. Carretera Gustavo Enrique Astiazarán Rosas, No. 46, Col. La Victoria, Hermosillo 83304, Mexico; jayala@ciad.mx

**Keywords:** Langmuir balance, nanotechnology, anti-quorum-sensing agents, plant compounds, bacterial biofilms

## Abstract

*Pseudomonas aeruginosa* infections have become more challenging to treat and eradicate due to their ability to form biofilms. This study aimed to produce hydrophobic nanoparticles by grafting 11-carbon and three-carbon alkyl chains to a chitosan polymer as a platform to carry and deliver carvacrol for improving its antibacterial and antibiofilm properties. Carvacrol–chitosan nanoparticles showed ζ potential values of 10.5–14.4 mV, a size of 140.3–166.6 nm, and an encapsulation efficiency of 25.1–68.8%. Hydrophobic nanoparticles reduced 46–53% of the biomass and viable cells (7–25%) within *P. aeruginosa* biofilms. Diffusion of nanoparticles through the bacterial biofilm showed a higher penetration of nanoparticles created with 11-carbon chain chitosan than those formulated with unmodified chitosan. The interaction of nanoparticles with a 50:50 *w*/*w* phospholipid mixture at the air–water interface was studied, and values suggested that viscoelasticity and fluidity properties were modified. The modified nanoparticles significantly reduced viable *P. aeruginosa* in biofilms (0.078–2.0 log CFU·cm^−2^) and swarming motility (40–60%). Furthermore, the formulated nanoparticles reduced the quorum sensing in *Chromobacterium violaceum*. This study revealed that modifying the chitosan polarity to synthesize more hydrophobic nanoparticles could be an effective treatment against *P. aeruginosa* biofilms to decrease its virulence and pathogenicity, mainly by increasing their ability to interact with the membrane phospholipids and penetrate preformed biofilms.

## 1. Introduction

*Pseudomonas aeruginosa* is a Gram-negative opportunistic bacterium that causes chronic lung infections in patients with cystic fibrosis, ventilator-associated respiratory diseases, and catheter-associated urinary tract infections [1]. *P. aeruginosa* has become more challenging to treat and eradicate due to its capacity to resist antibiotics [2,3,4]. *P. aeruginosa* infections involve biofilm formation and the presence of embedded persistent multidrug-tolerant cells [3,5]. Biofilms are surface-attached communities of bacterial cells enclosed in a matrix of self-produced extracellular polymeric substances (EPS) composed of carbohydrates, proteins, and nucleic acids [5]. EPS form a highly complex network with a three-dimensional structure that supports cellular attachment and acts as a protective barrier against conventional antibiotics [3,6,7]. Therefore, the development of strategies to address the global resistance to antimicrobial drugs is urgently required. Recently, there has been a surge in scientific interest in the development of agents with effective antibiofilm properties that affect bacterial viability, attenuate motility, cellular adhesion, and quorum sensing (QS) [8,9].

Carvacrol is a monoterpenoid phenol extracted from oregano and thyme essential oils used as a food flavoring [10]. Carvacrol has been recognized as an effective antimicrobial with a broad spectrum against Gram-positive and Gram-negative bacteria, including *Pectobacterium carotovorum*, *Escherichia coli*, *Salmonella* Typhimurium, *Staphylococcus aureus*, *Listeria monocytogenes*, and *P. aeruginosa* [11,12,13,14,15]. It can also decrease bacterial virulence factors such as motility, biofilm formation, and quorum sensing, which could aid in treating microbial infections [13,15]. This could allow its use to treat different infections due to its ability to inhibit oral, gastrointestinal, respiratory, and urinary tract pathogens. Some studies reported that carvacrol reduced the viability, metabolic activity, and biofilm formation of dental caries caused by *S. mutans* at a concentration of 100 µg/mL [16]. Moreover, carvacrol showed a 97% and 91% inhibition against *P. aeruginosa* adherence and biofilm formation, respectively [17]. This terpene at 150 and 450 µg/mL reduced adhesion, motility, and invasion of uropathogenic *E. coli* [18]. Similarly, carvacrol at 0.33 mg/mL was effective against *Salmonella* growth and attachment. The antimicrobial properties of carvacrol have been exploited in different industries including food, cosmetics, and medical. However, carvacrol has low aqueous solubility, a volatile nature, and poor bioavailability, which precludes its use as an antimicrobial and antibiofilm agent [11,19]. A promising strategy to overcome these limitations is incorporating nanostructured systems to improve their physicochemical properties and antibacterial activities [20].

Nanotechnology has a considerable research interest for its potential use as a drug delivery system in biomedicine, pharmaceutics, agriculture, and food, while reducing bacterial infections related to biofilms [21,22]. Chitosan nanoparticles with different bioactive compounds have been widely employed due to several benefits such as biocompatibility, high biodegradability, low toxicity, and great effectivity [20]. This biopolymer has been used to encapsulate essential oils and other plant-derived compounds [23]. For example, Tran and Hadinoto [24] managed to encapsulate quercetin into chitosan nanoparticles; this treatment improved the quercetin solubility in physiological fluids and reduced *P. aeruginosa* biofilm formation by reducing motility and quorum-sensing activities. Therefore, the revised evidence showed the potential use of chitosan nanoparticles with carvacrol as an antibiofilm treatment of *P. aeruginosa*. However, chemical modification of chitosan can be beneficial in improving its physicochemical properties. The primary amino group (–NH_2_) of the chitosan is relatively easy to modify due to the electron-donating effect with various functional groups such as carboxylic acid or thiol groups to facilitate diffusion within the biofilm and embedded cells.

Li, Yeh [25] revealed that positively charged quantum dots (QD) fully penetrated biofilms compared to anionic and neutral nanoparticles. These authors also reported that cationic and hydrophobic nanoparticles showed a higher and more homogeneous dispersion throughout the biofilm than cationic and hydrophilic structures. Similarly, Shebl, Farouk [26] prepared magnetic nanoparticles and modified their surface with different chemical groups to change hydrophobicity and surface charge parameters. They showed that the functional surface groups that produced positive charge or imparted hydrophobicity in the nanoparticles potentiated their antibacterial and antibiofilm activity.

These studies suggested that surface positivity and hydrophobicity can enhance nanoparticles’ antibacterial and antibiofilm activity. With this evidence, this study hypothesizes that the chemical modification of chitosan through the incorporation of alkyl chains is helpful to encapsulate carvacrol, enhancing its penetration in bacterial biofilms and favoring its interaction with bacterial membranes [27]. In this context, this study aimed to evaluate two different alkyl chains grafted onto a chitosan backbone (11-carbon and three-carbon length) to formulate and characterize carvacrol-loaded chitosan nanoparticles and determinate their effect on *P. aeruginosa* biofilms.

## 2. Results and Discussion

### 2.1. Chitosan Modification

**Figure 1:**
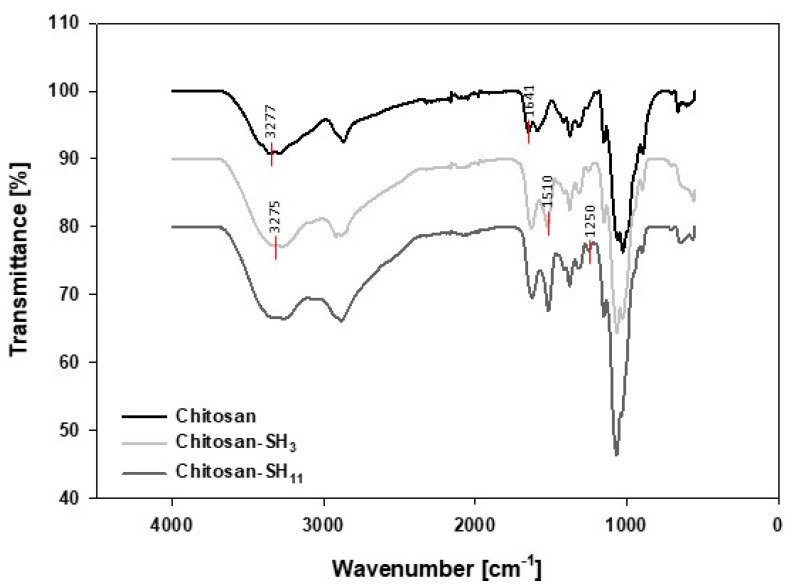
FTIR spectrum of chitosan, chitosan-SH_3_, and chitosan-SH_11_ polymer.

The chemical modification of the chitosan structure by adding three- and 11-carbon alkyl chains using mercaptopropionic and mercaptoundecanoic acid, respectively, was confirmed by FTIR-ATR (Figure 1). The FTIR spectra showed a set of characteristic bands of native chitosan at 1641 cm^−1^ and 1510 cm^−1^ assigned to the C=O stretching frequency and N–H groups (amine and amide II), respectively. The peak at 3277 cm^−1^ was associated with stretching the –NH_2_ and –OH groups. As seen in the modified chitosan spectrum, the band presence at a signal deformation 3225 cm^−1^ (–NH stretching amide) confirmed the amide bond formation (C–NH). The peak at 1250 cm^−1^ was attributed to the –SH stretching, confirming the presence of thiols as a terminal group linked to the chitosan molecule [28].

### 2.2. Characterization of Nanoparticles

**Figure 2:**
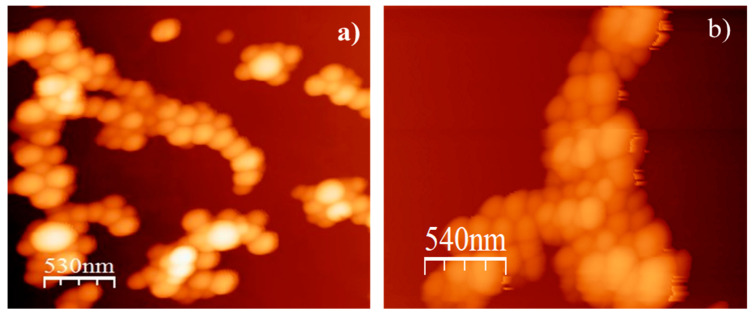
AFM images of (**a**) chitosan and (**b**) chitosan–SH_11_ nanoparticles synthesized by ionic gelation.

The nanoparticle characteristics (size, ζ potential, and encapsulation efficiency) are shown in Table 1. The average diameter of the carvacrol–chitosan, carvacrol–chitosan–SH_3_, and carvacrol–chitosan–SH_11_ particles was 140, 166, and 152 nm, respectively. The surface ζ potential of nanoparticles changed from +14.4 to +10.5 and +11.2 mV when using alkyl-modified chitosan. Figure 2 shows the AFM images of monodisperse particles using carvacrol–chitosan and carvacrol–chitosan–SH_11_ with spherical morphology and sizes between 130 nm and 170 nm. AFM images of the other nanoparticles are not shown because no morphological differences were observed. The encapsulation efficiency of carvacrol in the nanoparticles varied among native chitosan and modified chitosan. The highest entrapment efficiency (EE) in the nanoparticles was 68.8% when using chitosan–SH_11_.

The synthesis and modification of the chitosan nanoparticles were successful and in accordance with previous studies [28]. The electrostatic interaction between chitosan and TPP (sodium tripolyphosphate) developed the carvacrol nanoparticles. As expected, we could not observe a substantial change in the size of the nanoparticles after grafting the alkyl chains to native chitosan. However, the ζ potential values decreased using modified chitosan due to the reduction of amine groups during the modification process. As stated before, the alkyl chain grafted to the chitosan backbone did not significantly affect the size and shape of the nanoparticles observed in AFM images. The range of encapsulation efficiency of carvacrol in nanoparticles was in accordance with previous studies [20].

### 2.3. Antibacterial Activity of Nanoparticles against Planktonic P. aeruginosa

The minimum inhibitory concentration (MIC) of carvacrol reported previously by our group is 1.2 mg·mL^−1^ against *P. aeruginosa* (ATCC 10154) [29]. However, we could not find the MIC values for the nanoparticle treatments at the max concentration tested (2.4 mg·mL^−1^) in this study. Therefore, these concentrations were used in subsequent anti-virulence studies to ensure that loss of cell viability did not interfere with the evaluated responses.

### 2.4. Activity of Nanoparticles against Preformed Biofilms

The ability of nanoparticles to eradicate 24 h preformed mature biofilms of *P. aeruginosa* is observed in Table 2. Exposure of biofilms to 2.4 mg·mL^−1^ of carvacrol in the nanoparticles for 1 h reduced the *P. aeruginosa* biofilm mass formed in a microplate well and reduced the number of viable cells in preformed biofilms compared to the controls (*p* ≤ 0.05). In both assays, nanoparticles loaded with carvacrol showed a more significant effect than non-loaded nanoparticles. The most effective treatment to disrupt biofilm mass was the carvacrol–chitosan–SH_11_, which reduced up to 50% of the preformed biofilm, while carvacrol–chitosan–SH_3_ and carvacrol–chitosan achieved 46% biomass dispersion (*p* ≤ 0.05). The other treatments produced a reduction in biofilm biomass in the range 36–40%. Similarly, the carvacrol–chitosan–SH_11_ nanoparticles showed the most significant reduction (25%) in viable bacteria in preformed cells compared to control and other treatments with a reduction range of 7–14% (*p* ≤ 0.05).

The modified chitosan used to formulate carvacrol nanoparticles demonstrated biofilm eradication potential by decreasing biomass and viable cells. The nanoparticles effectively reduced the preformed biofilms after 1 h of contact, but total eradication could not be obtained at the tested concentrations, possibly due to the controlled release of carvacrol. It has been established that bioactive compounds could undergo different mechanisms to avoid biofilm formation and eliminate preformed biofilms. To disrupt a mature biofilm, the antimicrobial compounds should be able to penetrate the matrix of extracellular polymeric substances and damage the embedded viable cells through the disruption of cytoplasmic membranes or other mechanisms [30]. In this study, the eradication mechanism proposed for modified carvacrol nanoparticles is diffusion through the EPS matrix, reaching embedded cells and affecting their virulence.

### 2.5. Penetration of Nanoparticles into Biofilms Measured by Confocal Microscopy

**Figure 3:**
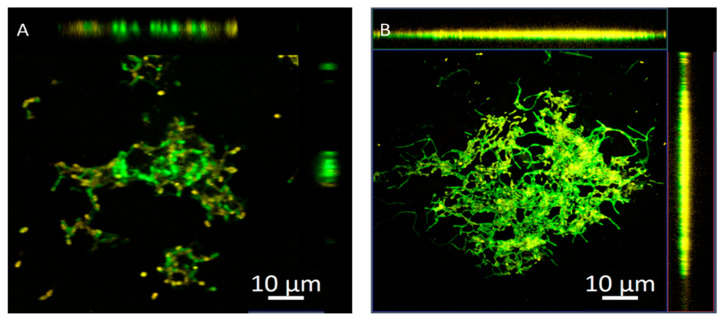
Representative 3D projection of image Z-stacks showing the distribution of bacterial cells (green) in *P. aeruginosa* biofilms and chitosan nanoparticles (orange): (**A**) chitosan; (**B**) chitosan–SH_11_.

Figure 3 shows the orthogonally overlaid images from a Z-stack to demonstrate the distribution of nanoparticles into biofilms. There was less fluorescence intensity deeper into the biofilms treated with chitosan nanoparticles. However, it remained on the surface as a lower overlay color of the two channels, suggesting that a positive charge is most suitable for interactions with an EPS matrix than penetrating a biofilm (Figure 3A). On the other hand, the chitosan–SH_11_ nanoparticles showed higher fluorescence intensities on both surfaces and in the middle regions of the biofilm at a 10 µm Z-stack (Figure 3B), suggesting a homogeneous distribution throughout the biofilm. Confocal laser scanning microscopy (CLSM) images of chitosan–SH_3_ nanoparticles were not shown because no difference was observed compared to chitosan nanoparticles.

Incorporating an alkyl chain into the chitosan polymer improved biofilm eradication activity by detaching biomass from polystyrene surfaces and reducing viable cells in biofilms compared to chitosan without modification. The confocal microscopic images demonstrated that chitosan–SH_11_ nanoparticles penetrated to a greater extent into the mature biofilms, indicating that the engineering of the surface by grafting alkyl chains into cationic polymers could achieve a better effect [31,32]. Previous studies have established that hydrophobic particles could penetrate bacterial biofilms. Li, Yeh [25] found that surface-engineering nanoparticles QDs can directly penetrate and distribute into *P. fluorescence* biofilms; they showed that anionic and neutral structures could not penetrate directly into the biofilm unlike their cationic counterpart. They also tested two cationic nanoparticles, one hydrophobic and another hydrophilic, observing that hydrophobic treatments are more regularly distributed throughout the biofilm.

### 2.6. Phospholipid Monolayer Interaction with the Formulated Nanoparticles

**Figure 4:**
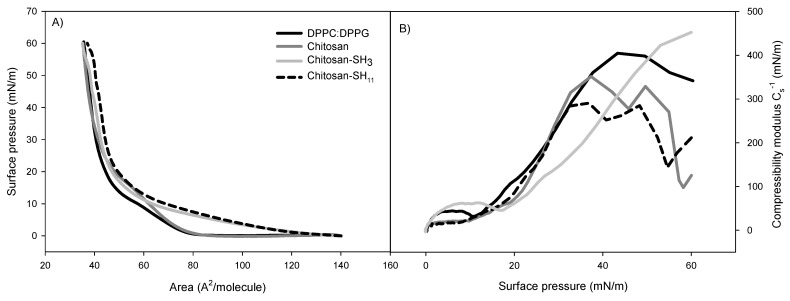
(**A**) Surface pressure–area (π-A) isotherms and (**B**) compressibility modulus (C_s_^−1^) values for the monolayers on subphases containing pure water with chitosan nanoparticles.

The nanoparticles were spread in the Langmuir trough subphase at a final concentration of 4 × 10^8^ particles·mL^−1^ at 25 ± 0.1 °C and compressed to obtain surface pressure–area isotherms (Figure 4A). The pure DPPC/DMPG monolayer isotherm showed a transition from typical liquid expanded (LE) to a more liquid condensed (LC) phase transition plateau at around 10 mN·m^−1^ and a solid phase at 35 Å^2^/molecule, indicating that phospholipids were packed. The introduction of nanoparticles into the lipidic monolayer shifted the isotherm, suggesting their interaction. The limiting area A_1_ increased from 86 Å^2^/molecule (absence of nanoparticles) to 87, 126, and 129 Å^2^/molecule when native chitosan, chitosan–SH_3_, and chitosan–SH_11_ nanoparticles were in the subphase, respectively, indicating the expansion of the DPPC/DMPG monolayer.

In addition, when modified chitosan nanoparticles were in the subphase, the distinctive plateau related to the phospholipid mix LE–LC coexistence region at 60 Å^2^/molecule was not observed. In contrast, when native chitosan nanoparticles were in the subphase, the LE–LC coexistence region could be observed with higher surface pressure (14.2 mN·m^−1^) than pure monolayer (9.8 mN·m^−1^). The effect of nanoparticle intercalation in the lipidic monolayer was also observed in lower molecular areas. In modified chitosan nanoparticles, the extrapolated area in the LC phase (A_0_) increased up to 55 Å^2^/molecule. This accomplished an approximate 10 Å^2^/molecule increment concerning the water subphase (45 Å^2^/molecule).

**Figure 5:**
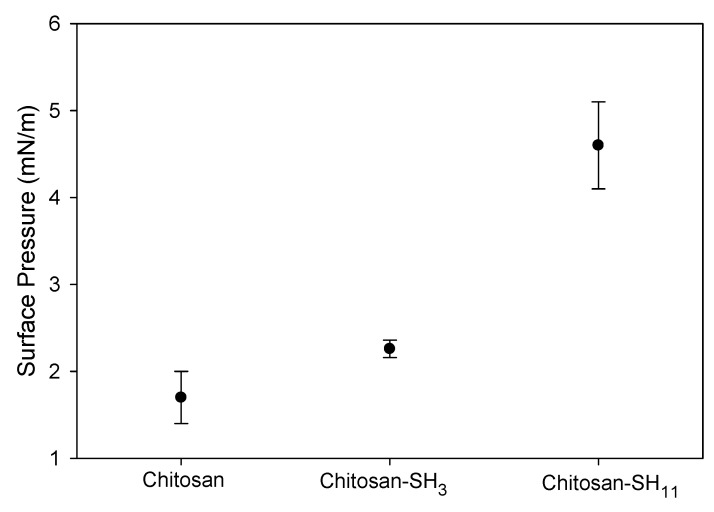
Surface pressure increment of DPPC/DMPG/nanoparticle monolayers at 30 mN·m^−1^ initial surface pressure of the DPPC/DMPG mixture obtained 4 h after injected nanoparticles in the pure water subphase (*n* = 3).

The changes previously described in the isotherms can be best studied by estimating the compressional modulus of the monolayer (C_s_^−1^) vs. surface pressure (Figure 4B). For the phospholipid mixture, a maximum value of C_s_^−1^ was observed at 405 mN·m^−1^, and a relative minimum was observed at 10.2 mN·m^−1^, which corresponds to the middle of the LE–LC equilibrium on the isotherm plateau. The relative maximum C_s_^−1^ value in the LC phase (just before collapsing) of DPPC/DMPG monolayers on subphases containing nanoparticles was 282 and 325 mN·m^−1^ for chitosan–SH_11_ and chitosan–SH_3_ nanoparticles, respectively. The nanoparticle penetration into preformed phospholipid monolayers at physiologically relevant surface pressures of 30 mN·m^−1^ is shown in Figure 5. After 4 h of pressure release, the highest increment value was 4.5 mN·m^−1^, followed by 2.3 and 1.8 mN·m^−1^, for chitosan–SH_11_, chitosan–SH_3_, and native chitosan, respectively, compared to the phospholipid pressure in the free nanoparticle subphase.

As the nanoparticles penetrate biofilms, they can exert antibacterial activity against the embedded viable cells. Therefore, we studied the interaction between the nanoparticles and phospholipid model membranes using Langmuir balance isotherms. The phospholipid monolayer isotherm at the air–water interface was in agreement with that already reported [33]. The adsorption of all nanoparticles caused an increase in surface pressure at a larger molecular area than the system without treatments determined by extrapolation at the intersection of the abscissa axis with the tangent of the isotherm when the pressure starts to rise. The compression isotherm DPPC/DPPG on the nanoparticle subphase shifted the isotherm, suggesting a reduced packing density of phospholipid monolayer. The increase in A_0_ values when the modified nanoparticles were at the subphase indicated the expansion of the DPPC/DMPG monolayer because of the hydrophobic and electrostatic interactions of the alkyl chain and the positive surface charge of modified chitosan nanoparticles.

Permeability characteristics of biological membranes are related to monolayer compressibility [34]. The decrease observed in the modulus is proportional to the increase in fluidity/elasticity of the monolayer [35]. The C_s_^−1^ values were reduced in the presence of nanoparticles in the subphase, suggesting that they disturbed the packing density of the phospholipid monolayers due to the hydrophobic and electrostatic interactions that could expand the monolayers [36].

Nanoparticle addition at the subphase showed an effect on the viscoelastic properties of phospholipidic monolayers due to a rise in membrane fluidity caused by a change in the molecular packaging and an alteration of the molecular area. According to the different phospholipid–nanoparticle isotherms and the results of their penetration into monolayers at 30 mN·m^−1^, we suggest that adding alkyl chains to the surface of the nanoparticles could have a better interaction with bacterial membranes. It seems that the hydrophobic interaction plays a more important role than just an electrostatic interaction between the cationic chitosan nanoparticles and the anionic nature of the membrane model, highlighting that hydrophobic interaction can enhance membrane and biofilm penetration. Although this monolayer represented the bacterial membrane in its planktonic state, other research articles confirmed that surface engineering of nanoparticles is essential as hydrophobicity can enhance biofilm penetration [37,38,39,40].

### 2.7. Antibiofilm Activity

All nanoparticles exhibited a significant reduction in biofilm formation in a range of 0.7 to 2 log CFU·cm^−2^ compared to untreated bacteria (Table 2). Chitosan nanoparticles reduced biofilm formation, but carvacrol-loaded nanoparticles showed a better effect (*p* ≤ 0.05). Out of all the nanoparticles samples, the carvacrol–chitosan–SH_11_ treatment had the most significant impact on biofilm formation, reducing 2 log CFU·cm^−2^ of adhered cells to the polystyrene surface (*p* ≤ 0.05), followed by carvacrol–chitosan–SH_3_ and carvacrol in native chitosan compared to control. Viable planktonic cells were counted to ensure that the effect of nanoparticles was on biofilm formation and not due to a reduction in viability. The chitosan nanoparticles without carvacrol did not affect *P. aeruginosa* viability, while the carvacrol in native chitosan nanoparticles produced slight changes in planktonic cell viability compared to control (range of reduction 0.23–0.34 log CFU·mL^−1^) (*p* ≤ 0.05).

The anti-virulence strategy is a promising approach to avoid antibiotic bacteria resistance by attenuating virulence factors such as biofilm formation without affecting cell viability [9]. All nanoparticles exhibited a significant reduction in biofilm formation. The antibiofilm effect of carvacrol in this study could be through multiple mechanisms. Carvacrol, at low concentrations, can react with bacterial proteins and reduce their attachment to surfaces. Furthermore, carvacrol can suppress motility, reduce the production of extracellular polymeric substances, and interfere with QS, thereby hindering the formation of bacterial biofilms [13,15,41].

The nanoparticles synthesized in this study could inhibit surface adhesion and biofilm formation, possibly due to their antibacterial effect associated with the interaction between the negative charge of the microbial membrane and the cationic charge of chitosan [42]. The nanoparticles formulated with modified chitosan with an alkyl chain of 11 carbons (–SH_11_) showed the highest reduction in biofilm formation, which agrees with the results observed in the Langmuir balance that indicated that these nanoparticles showed a more significant interaction with membrane phospholipids and could penetrate bacterial membranes more than unmodified chitosan and chitosan–SH_3_.

### 2.8. Antimotility Effect

**Figure 6:**
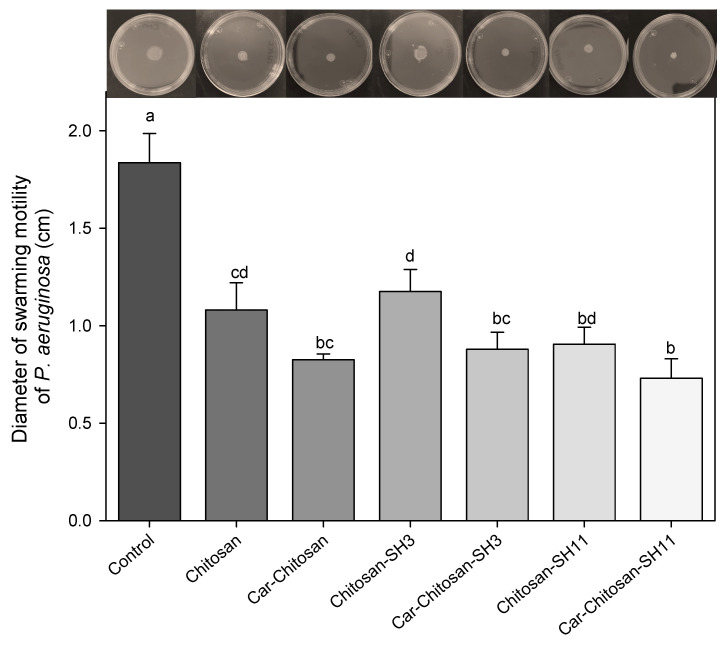
Swarming motility of *P. aeruginosa* exposed to different nanoparticles without and with carvacrol (Car) loaded in chitosan modified with three- (SH_3_) or 11-carbon (SH_11_) alkyl chains. Values are expressed as the mean ± standard deviation. Different letters indicate significant differences (*p* ≤ 0.05).

The motility results are shown in Figure 6. Only a small swarm movement around the bacterial growth could be observed in all treatments. The presence of nanoparticles reduced approximately 40–60% of swarming motility compared to control. The carvacrol–chitosan–SH_11_ nanoparticles obtained the more significant reduction with 60%, followed by carvacrol in native chitosan and carvacrol in chitosan–SH_3_ with a decrease of approximately 52–54%, although no significant difference was found.

One strategy proposed to decrease biofilm formation is to affect virulence factors involved in such process [7]. The swarming motility of *P. aeruginosa* plays an essential role in surface attachment at the early stages of biofilm formation [43]. Therefore, the reduction in *P. aeruginosa* motility could influence the removal of biofilm formation. In this study, all the evaluated treatments reduced swarming motility. The mechanism underlying how terpenoid compounds inhibit bacterial motility is still unclear; however, some authors proposed some approaches. Leja, Drożdżyńska [44] reported that essential oils could change the bacterial shape, resulting in the loss of motility in *Pseudomonas orientalis*. Furthermore, some authors reported the effectivity of carvacrol at subinhibitory concentrations to reduce *Campylobacter jejuni* motility due to a possible alteration in flagella function [45] or a reduction in the expression of motility-related genes such as *motA* [41].

Tran and Hadinoto [24] proposed a similar approach to our study. They used quercetin due to its excellent antimicrobial effects and limited use as a treatment due to its low solubility. Therefore, they developed a nanocomplex of chitosan with quercetin to improve the solubility in physiological fluids. They described that subinhibitory concentrations of quercetin in the nanocomplex produced lower rates of biofilm formation and a 40% reduction in motility of *P. aeruginosa* compared to control and native quercetin. Furthermore, a synergistic effect was observed among chitosan and quercetin due to a higher quercetin exposure because of its greater solubility [24]. Mohammed, Rayyif [46] created a nanostructured system using Fe_3_O_4_ nanoparticles and eugenol, a natural plant compound previously shown to inhibit microbial virulence. When used at subinhibitory concentrations, the hybrid nanosystem was able to modulate attachment, biofilm development, and the production of soluble virulence enzymes.

### 2.9. Anti-Quorum-Sensing Effect

**Figure 7:**
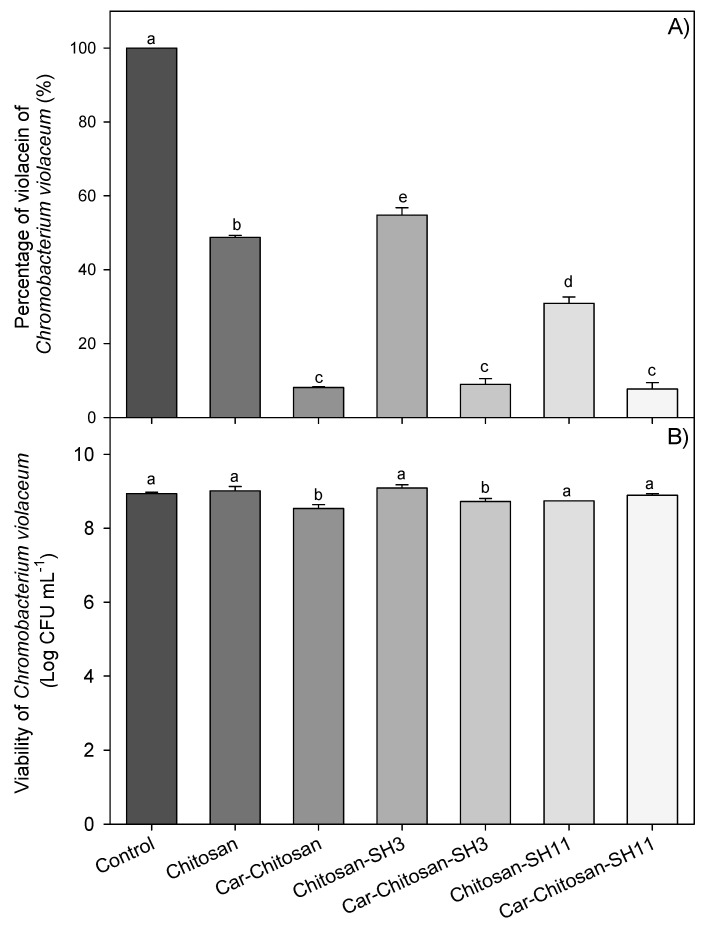
(**A**) Inhibition of violacein production and (**B**) cell viability of *C. violaceum* exposed to different nanoparticles without and with carvacrol (Car) loaded in chitosan modified with three- (SH_3_) or 11-carbon (SH_11_) alkyl chain. Values are expressed as the mean ± standard deviation. Different letters indicate significant differences (*p* ≤ 0.05).

To assess the effect of nanoparticles in quorum-sensing inhibition, *Chromobacterium violaceum* CV026 was used as a biosensor bacterial model which produces violacein in response to quorum sensing. All nanoparticles tested significantly reduced violacein production without affecting the cell viability of *C. violaceum* compared to control (Figure 7). Nanoparticles with carvacrol were more effective in lowering pigment production than nanoparticles without this compound. Carvacrol–chitosan–SH_11_, carvacrol–chitosan–SH_3_, and carvacrol–chitosan nanoparticles showed a 92% pigment reduction. The alkylation of chitosan did not show a significant difference (*p* > 0.05) in the QS test when loaded with carvacrol; however, carvacrol–chitosan–SH_11_ inhibited 30% more violacein production than carvacrol–chitosan–SH_3_ nanoparticles.

QS inhibition could attenuate bacterial pathogenicity and biofilm formation. All nanoparticles tested significantly reduced violacein production without affecting the cell viability of *C. violaceum* compared to control. A previous study showed that pure carvacrol at 1.2 mg·mL^−1^ decreased violacein production in *C. violaceum* by approximately 50% [29], while, in our study, the encapsulated carvacrol reduced more pigment production at the same concentration. This effect could be attributed to the anti-quorum-sensing activity of chitosan [24] and carvacrol by interfering with lactones, synthase CviI, receptor CviR, or related gene expression. It has been reported that carvacrol reduces biofilm formation and motility through QS inhibition [15]. Carvacrol (1.2 mg·mL^−1^) reduced 60% of acyl-homoserine lactones (signaling molecule) of *P. aeruginosa,* suggesting a reduction in LasI synthase activity. Moreover, the gene expression of *lasI* (autoinducer synthase) was not affected, while the expression of *lasR* (autoinducer receptor) was reduced by carvacrol, indicating the reduction of signaling molecules could be caused the gene expression reduction [15]. Another study using terpenes demonstrated that nanostructured lipid carriers containing α-terpineol downregulated more efficiently the QS genes (lasI, lasR, rhlI, and rhlR) of *P. aeruginosa* than the free α-terpineol [47].

Chitosan has been widely employed to encapsulate other bioactive compounds to inhibit bacterial QS. Cinnamaldehyde loaded into chitosan nanoparticles demonstrated anti-QS activity by reducing QS-regulated virulence factors related to biofilm formation. The nanoparticles showed a mean particle size of 208.12 nm with an encapsulation efficiency of 65%. Furthermore, sublethal concentrations of cinnamaldehyde altered the *P. aeruginosa* QS activity, observing a decrease in LasA staphylolytic activity when exposed to cinnamaldehyde–chitosan nanoparticles. Moreover, these authors associated the anti-QS effect with the reduction in swarming and swimming motility and the removal of extracellular polymeric substances of *P. aeruginosa* [48]. Similarly, kaempferol–chitosan nanoparticles with a size of 192 nm and encapsulation efficiency of 78–93% showed strong and long-term anti-QS activity with a reduction in violacein production of up to 76% *C. violaceum* CV026 compared to control and pure kaempferol [20]. The studies mentioned above highlight the need to employ nanosystems with natural compounds to enhance their bioactive properties.

## 3. Materials and Methods

### 3.1. Chitosan Modification

Low-molecular-weight chitosan (100,000 g·mol^−1^) with a degree of deacetylation of 75–85% and viscosity of 0.02–0.3 Pa·s, 3-mercaptopropionic acid (SHCH_2_CH_2_CO_2_H), and 11-mercaptoundecanoic acid (SH(CH_2_)_10_CO_2_H) were purchased from Sigma Aldrich (St. Louis, Mo, USA) and used in this experiment. The modification of chitosan was achieved by forming an amide bond between the polymer –NH_2_ groups and the –COOH of mercaptopropionic acid and mercaptoundecanoic acid to graft a three- and 11-carbon alkyl chain, respectively, with a 10% substitution degree for both cases. Specifically, a chitosan solution was formulated by dissolving 1.5 g in 150 mL of HCl (0.1 M). Next, a solution of each alkyl chain substitute (0.0208 mM), EDAC (*N*-(3-dimethylaminopropyl)-*N*′-ethylcarbodiimide hydrochloride, 0.104 mM), and NHS (*N*-hydroxysuccinimide, 0.104 mM) in dimethylformamide (DMF, 15 mL) was prepared. Then, the chitosan solution (1% *w*/*v*) was added dropwise to the DMF solution, with continued stirring for 24 h, and dialyzed for 3 days.

### 3.2. Fourier-Transform Infrared Spectroscopy (FT-IR)

FT-IR spectra were recorded on the spectrometer Spectrum Two (Perkin-Elmer, Llantrisant, UK) equipped with a single diamond accessory of attenuated total reflectance (ATR) at room temperature with the purpose of evidencing physicochemical changes in the modified chitosan. The spectrum was recorded at the range 4000–350 cm^−1^ at a resolution of 1 cm^−1^. Chitosan was analyzed following a freeze-drying process before and after derivatization as previously reported by our group [49,50,51].

### 3.3. Synthesis of Nanoparticles

Nanoparticles were produced by the ionic gelation method using the crosslinker TPP as previously reported [51]. Carvacrol (>98%, Sigma Aldrich) emulsion was formulated by adding 25 mg to 10 mL of Tween-80 aqueous solution (1% *v*/*v*) at 25 °C; this mixture was then sonicated for 2 min at 50% amplitude (Q500 sonicator) to form an emulsion. Previously, 50 mg of each chitosan was dispersed in 100 mL of acetic acid (1% *v*/*v*) with magnetic agitation overnight at 300 rpm at room temperature to ensure its complete dissolution (Thermo Fisher Scientific, Waltham, MA, USA). The carvacrol emulsion (2.04 mL) was added to this solution to achieve a weight ratio of 1:1 *v*/*v* (chitosan/carvacrol). Subsequently, the solution was deposited in a water bath at 60 °C for 10 min and then transferred to a water bath at 4 °C, followed by the immediate addition of 2 mL of TPP solution (2 mg·mL^−1^); this reaction was carried out for 20 min. The resulting nanoparticles were recovered by centrifugation at 9000 rpm for 30 min and were finally resuspended in deionized water.

### 3.4. Carvacrol Content in the Chitosan Nanoparticles

The carvacrol content encapsulated in the chitosan nanoparticles was quantified by HPLC as previously described [52] with some modifications.. Chromatographic analyses were carried out with an HPLC 1200 system (Agilent Technologies, Santa Clara, CA, USA) using a Zorbax Eclipse XDB-C18 (80 Å 5 µm, 4.6 × 250 mm) reversed-phase column at 25 °C. The evaluation was carried out using an isocratic mode (water and acetonitrile, 50/50 (*v*/*v*)) at a 1 mL·min^−1^ flow rate for 10 min and a 10 µL stainless-steel sample loop. Quantifications were performed at 242 nm using an external calibration curve as previously reported [49]. The carvacrol entrapment efficiency (EE%) was calculated as

EE% = (carvacrol_total_ − carvarcol_supernatant_/theoretical carvacrol content) × 100. (1)

### 3.5. Zeta Potential, Size, and Morphology of the Nanoparticles

The zeta potential (ζ) was evaluated by microelectrophoresis, and the particle size was measured via dynamic light scattering utilizing a Zetasizer Nano ZS90 (Malvern Instruments, Worcestershire, UK). The nanoparticles were suspended in Milli-Q water and slightly agitated to spread them. The experiment was conducted in triplicate at 25 °C; and size results were expressed in nanometers (nm), and the ζ potential was expressed in millivolts (mV). The nanoparticle morphology was observed using atomic force microscopy (AFM, model JSPM-4210, JEOL, Tokyo, Japan) in noncontact mode using an NSC15 silicon cantilever (MikroMasch, Portland, OR, USA) and analyzed with WSxM software.

### 3.6. Antibacterial Activity of the Carvacrol–Chitosan Nanoparticles

The antibacterial effect of the nanoparticles was assessed against *Pseudomonas aeruginosa* (ATCC 10154) using the micro-well dilution assay. For this, 5 µL of a 19 h bacterial culture (1 × 10^8^ CFU·mL^−1^) in Luria–Bertani (LB) broth was placed in a sterile 96-well microtiter plate (Costar 96), followed by dilutions of each treatment (295 µL) (chitosan, chitosan–carvacrol, chitosan–SH_3_, carvacrol–chitosan–SH_3_ nanoparticles, chitosan–SH_11_, carvacrol–chitosan–SH_11_ nanoparticles) in LB broth. The carvacrol content in the nanoparticles dispersed in the medium ranged from 0.3–2.4 mg·mL^−1^. The plate was then incubated at 37 °C for 24 h, and the minimum inhibitory concentration was determined visually when no growth was observed [53].

### 3.7. Biofilm Eradication Activity of Nanoparticles

The effect of carvacrol–chitosan nanoparticles on eradication of *P. aeruginosa* biofilm was evaluated by counting viable cells in exposed preformed biofilms and by determining biofilm disruption according to absorption of crystal violet assay [54]. The objective of these techniques was to evaluate the capacity of the nanoparticles to eliminate formed biofilms; in the future, this could be contemplated as a treatment once the disease has appeared. Biofilms were formed in polystyrene surface coupons (1 × 1 × 0.1 cm) contained in LB broth (5 mL) by the inoculation with 19 h bacteria culture (1 × 10^6^ CFU·mL^−1^). These tubes were incubated at 37 °C for 24 h; subsequently, the coupons were gently washed with sterile water and exposed for 1 h to different treatments, reaching 2.4 mg·mL^−1^ carvacrol content. Then, coupons were rinsed in saline solution (5 mL) and subjected to an ultrasound bath (Branson 2510, Branson Ultrasonics, Danbury, CT, USA) at 40 kHz for 5 min. Subsequently, viable cells were counted in the serial dilutions by plating in LB agar at 37 °C for 24 h. Results were stated as the number of survival bacteria per area (CFU·cm^−2^).

For the biofilm disruption assay, 5 µL of an 18 h inoculum of *P. aeruginosa* (1 × 10^8^ CFU·mL^−1^) and 295 µL of LB broth were put into a sterile 96-well polystyrene microplate and incubated at 37 °C for 24 h. After that time, the microplate was softly washed with sterile distilled water to eliminate the culture medium and unattached cells. Later, 300 µL of each treatment dissolved in LB broth was added to the microplate for 1 h. The nanoparticles doses were adjusted to obtain a similar carvacrol content (2.4 mg·mL^−1^). The culture was removed, and the wells were washed with distilled water and then allowed to dry for 15 min. The adhered cells to the polystyrene material were stained with 150 µL of crystal violet (0.1% *w*/*v*) for 45 min. Crystal violet dye was removed by washing the microplate and dried for 15 min. Then, 150 µL of acetic acid (33%) was added to dilute the adhered dye in each well and allowed to stand for 15 min. Successively, 150 µL of each sample was placed in another microplate, and the OD was assessed at 600 nm in a FLUOstar Omega spectrophotometer (BMG Labtech, Chicago, IL, USA). A well with only LB broth was used as a blank, and the bacteria without nanoparticles treatment were used as a control. The biomass of control bacteria was contemplated as 100%, and results were indicated as the percentage of biomass production (%) compared to the control. All experiments were performed in triplicate.

### 3.8. Confocal Laser Scanning Microscopy (CLSM)

Rhodamine B was used to label the nanoparticles to evaluate their diffusion and distribution into *P. aeruginosa* biofilms as previously described [55]. For this, 5 mL of Rhodamine B solution (0.1 mg·mL^−1^) was mixed with the chitosan solution before adding TPP; the remaining procedure was the same as described earlier for the nanoparticles synthesis. This experiment was only performed with native chitosan and chitosan–SH_11_ nanoparticles because no difference was observed in biofilm eradication between chitosan and chitosan–SH_3_ nanoparticles. Chitosan and chitosan–SH_11_ nanoparticles were added to 24 h aged bacteria biofilm adhered to glass coverslips for 1 h. The biofilms were stained with Syto9 and fixed for 30 min, before washing multiple times, and CLSM images were obtained using two fluorescent channels to detect bacterial cells (Cyto9, 483/503 Em/Ex) and chitosan nanoparticles (Rhodamine B, 533/627 Em/Ex).

### 3.9. Isotherms of Phospholipids Monolayer with Nanoparticles

The interaction of the chitosan nanoparticles with membrane models was evaluated using a Langmuir balance (Nima Technologies, Ltd., Coventry, UK) with a Langmuir Blodgett (Model 611). A phospholipid mixture of DPPC and DMPG (dipalmitoylphosphatidylcholine and 1,2-dimyristoyl-*sn*-glycerol-3-phosphoglycerol, respectively) was used to obtain the surface pressure–area (π–A) isotherms with a surface tension precision of 0.1 mN·m^−1^. First, the phospholipids were prepared in chloroform (1 mg·mL^−1^) for a final ratio of 1:1 *v*/*v*. Next, the phospholipid mix (20 µL) was spread onto the subphase (250 mL of water) using a Hamilton microsyringe. Following 1 h of solvent evaporation, nanoparticles were injected in the subphase, and, after pressure stabilization, the isotherms were achieved at a 20 cm^2^·min^−1^ barrier speed. The same concentration of treatments was used for each modified chitosan. The nanoparticle concentration per mL was determined by their relative viscosity, as previously reported [56]. The experiment was conducted in a glass cabin free of dust at 25 °C and repeated three times. In addition, the monolayer isothermal compressibility modulus (C_s_^−1^) at a provided pressure (π) was analyzed to identify the interfacial elasticity values and was obtained by applying the following equation: C_s_^−1^ = A (δπ δA^−1^) [57].

### 3.10. Penetration of Nanoparticles into the Phospholipid Monolayers

The phospholipid monolayer at the air–water interphase was compressed at 30 mN·m^−1^ at a 50 cm^2^·min^−1^ speed to test the penetration of the carvacrol chitosan nanoparticles into the membrane model. Briefly, the pressure was allowed to stabilize for 15 min, and then the nanoparticle solution (5 mL, 1 × 10^11^ particles·mL^−1^) was introduced in the subphase, and the pressure was released. Finally, the increase in surface pressure control was evaluated for 4 h [36]. The experiment was conducted in triplicate.

### 3.11. Antibiofilm Activity of Carvacrol–Chitosan Nanoparticles

This technique aimed to evaluate the nanoparticle capacity to avoid the formation of biofilms, acting as a preventive treatment. The effect of nanoparticles on *P. aeruginosa* biofilm formation was carried out by counting the attached cells on polystyrene coupons (1 × 1 × 0.1 cm) as previously described [29]. First, nanoparticles were diluted in tubes containing LB broth (5 mL) and the polystyrene coupons for the assay. Nanoparticle doses were adjusted to obtain a similar carvacrol content (1.2 mg·mL^−1^). Then, the tubes were inoculated with a bacteria culture (1 × 10^6^ CFU·mL^−1^) and incubated at 37 °C for 24 h. Next, coupons were separated and washed thrice with sterile saline solution to remove all the nonattached cells. Then, coupons were added to 5 mL of sterile saline solution and sonicated using an ultrasonic bath (Branson 2510, Branson Ultrasonics, Danbury, CT, USA) at 40 kHz for 5 min to remove the attached bacteria. Subsequently, serial dilutions were made of cells suspensions, plated in LB agar, and incubated at 37 °C for 24 h. The results were quantified as the number of adhered cells by area (log CFU·cm^−2^), and all tests were conducted in triplicate. In addition, viable planktonic cells were counted in LB agar to ensure that the effect of the nanoparticles was on the biofilm formation.

### 3.12. Motility of P. aeruginosa Exposed to Nanoparticles

The swarming motility of *P. aeruginosa* treated with carvacrol–chitosan nanoparticles was evaluated [15]. For this, 20 µL of a bacterial inoculum was grown in LB broth in the presence of different treatments of carvacrol chitosan nanoparticles at 37 °C for 24 h. Treatment doses were adjusted to obtain a similar carvacrol content (1.2 mg·mL^−1^). The bacteria without exposure to the compounds were taken as a control. Then, the exposed bacteria (10 µL, 1 × 10^6^ CFU·mL^−1^) were inoculated in the center of LB semisolid plates (0.5% agar) and incubated at 37 °C for 24 h. The results were expressed as mm, and all tests were conducted in triplicate.

### 3.13. Anti-Quorum-Sensing Effect of Carvacrol Nanoparticles

The anti-quorum-sensing activity of carvacrol-loaded chitosan nanoparticles was evaluated using *Chromobacterium violaceum* (ATCC 12472), a biosensor model that produces a purple pigment (violacein) as an indicator of cellular communication [29]. First, nanoparticles were serially diluted in LB broth (1.2 mg·mL^−1^ carvacrol content), inoculated with a 19 h culture of *C. violaceum* (1 × 10^6^ CFU·mL^−1^), and incubated at 30 °C for 24 h. After that time, each sample (1 mL) was centrifuged (15,800× *g* for 10 min) to precipitate violacein. Next, the supernatant was removed, and the pellet was diluted in dimethyl sulfoxide (1 mL) and agitated until it completely dissolved. These solutions were centrifuged (15,800× *g* for 10 min) twice to precipitate bacterial cells, and the supernatant was used to quantify the violacein production by quantifying the optical density (OD) at 585 nm in a microplate reader (Fluostar Omega BMG Labtech, Chicago, IL, USA). The results were indicated as a percentage of violacein production considering that 100% of pigment production was the control bacteria without nanoparticles. Furthermore, the cell viability of *C. violaceum* was analyzed to ensure that the pigment reduction was not attributed to a decrease in cell viability. For this, 1 mL of each sample was serially diluted in saline solution, plated in LB agar, and incubated at 30 °C for 24 h. The results were expressed as log CFU·mL^−1^, and the experiment was done in triplicate.

### 3.14. Statistical Analysis

A completely randomized experimental design was done for all experiments. An analysis of variance (ANOVA) followed by the Tukey–Kramer multiple comparison test was used to estimate significant differences between treatments (*p* ≤ 0.05) using the Statistical Number Crunch System Software (NCSS, 2011).

## 4. Conclusions

This study highlighted the potential of carvacrol loaded into chitosan nanoparticles as an effective treatment for *P. aeruginosa* biofilm-related infections. The alkylation of chitosan nanoparticles was studied for their ability to eradicate biofilms and avoid biofilm formation. The results showed that incorporating an alkyl chain of 11 carbons to chitosan produced a more significant effect to diffuse throughout *P. aeruginosa* biofilms, disrupting biofilm biomass and reducing viable cells in biofilms. To explain the viable cell reduction, we studied the nanoparticle interaction with a membrane model using the Langmuir balance technique. Carvacrol–chitosan–SH_11_ nanoparticles disturbed the packing density of the phospholipid monolayers, changing the compression isotherms in the subphase and inducing a higher expansion of the membrane model. Moreover, this study demonstrated that carvacrol trapped in chitosan grafted with two different alkyl chain lengths reduced biofilm formation and motility of *P. aeruginosa* and reduced violacein pigment production of the reporter strain *C. violaceum*. Further research should study the controlled release of carvacrol from these nanoparticles and their in vivo effects in animal models.

## Figures and Tables

**Table 1:** Encapsulation efficiency and overall properties of carvacrol–chitosan nanoparticles.

Formulation	Size (nm)	PDI *	ζ Potential (mV)	% Carvacrol EE *
Chitosan	140.3 ± 1.3	0.2	14.4	50.7 ± 1
Chitosan–SH_3_	166.6 ± 5.1	0.1	10.5	25.1 ± 4.7
Chitosan–SH_11_	152.1 ± 2.1	0.3	11.2	68.8 ± 3.2

Means ± standard deviations are illustrated * PDI: polydispersity, * EE: entrapment efficiency.

**Table 2:** Effect of different chitosan nanoparticles on biofilm formation in polystyrene surfaces and percentage eradication of preformed *Pseudomonas aeruginosa* biofilms (24 h, 37 °C).

Nanoparticles Treatments	Bacterial Adhered Cells in Polystyrene Surface (log CFU·cm^−2^)	Percentage of Biofilm Biomass (%)	Viable Bacteria Cells in Preformed Biofilms (log CFU·cm^−2^)
Control	7.82 ± 0.01 ^a^	100	7.09 ± 0.03 ^a^
Chitosan	7.04 ± 0.03 ^bc^	59.91 ± 0.40 ^a^	6.60 ± 0.05 ^b^
Carvacrol–chitosan	6.03 ± 0.07 ^d^	53.08 ± 0.51 ^bc^	5.92 ± 0.02 ^de^
Chitosan–SH_3_	7.16 ± 0.02 ^b^	63.76 ± 1.76 ^d^	6.39 ± 0.09 ^bc^
Carvacrol–chitosan–SH_3_	6.09 ± 0.05 ^d^	53.30 ± 0.71 ^c^	5.81 ± 0.07 ^e^
Chitosan–SH_11_	6.89 ± 0.07 ^c^	61.62 ± 1.60 ^d^	6.16 ± 0.15 ^cd^
Carvacrol–chitosan–SH_11_	5.82 ± 0.06 ^e^	46.37 ± 1.23 ^b^	5.37 ± 0.19 ^f^

Means for three independent experiments ± standard deviations are illustrated. Different letters indicate significant differences (*p* ≤ 0.05) among treatments.

## Data Availability

The data presented in this study are available on request from the corresponding author.

## Sample Availability

Samples of the compounds are available from the authors.

## References

[1] Ciofu O., Tolker-Nielsen T. (2019). Tolerance and resistance of *Pseudomonas aeruginosa* biofilms to antimicrobial agents—How *P. aeruginosa* can escape antibiotics. Front. Microbiol..

[2] Pachori P., Gothalwal R., Gandhi P. (2019). Emergence of antibiotic resistance *Pseudomonas aeruginosa* in intensive care unit; a critical review. Genes Dis..

[3] Pang Z., Raudonis R., Glick B.R., Lin T.-J., Cheng Z. (2019). Antibiotic resistance in *Pseudomonas aeruginosa*: Mechanisms and alternative therapeutic strategies. Biotechnol. Adv..

[4] Hirsch E.B., Tam V.H. (2010). Impact of multidrug-resistant *Pseudomonas aeruginosa* infection on patient outcomes. Expert. Rev. Pharmacoecon. Outcomes Res..

[5] Maurice N.M., Bedi B., Sadikot R.T. (2018). *Pseudomonas aeruginosa* biofilms: Host response and clinical implications in lung infections. Am. J. Respir. Cell Mol. Biol..

[6] Jakobsen T.H., Bjarnsholt T., Jensen P.Ø., Givskov M., Høiby N. (2013). Targeting quorum sensing in *Pseudomonas aeruginosa* biofilms: Current and emerging inhibitors. Future Microbiol..

[7] Mauch R.M., Jensen P.Ø., Moser C., Levy C.E., Høiby N. (2018). Mechanisms of humoral immune response against *Pseudomonas aeruginosa* biofilm infection in cystic fibrosis. J. Cyst. Fibros..

[8] Ciofu O., Tolker-Nielsen T., Jensen P.Ø., Wang H., Høiby N. (2015). Antimicrobial resistance, respiratory tract infections and role of biofilms in lung infections in cystic fibrosis patients. Adv. Drug Deliv. Rev..

[9] Maura D., Ballok A.E., Rahme L.G. (2016). Considerations and caveats in anti-virulence drug development. Curr. Opin. Microbiol..

[10] Suntres Z.E., Coccimiglio J., Alipour M. (2015). The bioactivity and toxicological actions of carvacrol. Crit. Rev. Food Sci. Nut..

[11] Cacciatore F.A., Dalmás M., Maders C., Isaía H.A., Brandelli A., da Silva Malheiros P. (2020). Carvacrol encapsulation into nanostructures: Characterization and antimicrobial activity against foodborne pathogens adhered to stainless steel. Food Res. Int..

[12] Giovagnoni G., Rossi B., Tugnoli B., Ghiselli F., Bonetti A., Piva A., Grilli E. (2020). Thymol and carvacrol downregulate the expression of *Salmonella typhimurium* virulence genes during an in vitro infection on caco-2 cells. Microorganisms.

[13] Gutierrez-Pacheco M.M., Gonzalez-Aguilar G.A., Martinez-Tellez M.A., Lizardi-Mendoza J., Madera-Santana T.J., Bernal-Mercado A.T., Vazquez-Armenta F.J., Ayala-Zavala J.F. (2018). Carvacrol inhibits biofilm formation and production of extracellular polymeric substances of *Pectobacterium carotovorum* subsp. carotovorum. Food Control.

[14] Niza E., Božik M., Bravo I., Clemente-Casares P., Lara-Sanchez A., Juan A., Klouček P., Alonso-Moreno C. (2020). PEI-coated PLA nanoparticles to enhance the antimicrobial activity of carvacrol. Food Chem.

[15] Tapia-Rodriguez M.R., Bernal-Mercado A.T., Gutierrez-Pacheco M.M., Vazquez-Armenta F.J., Hernandez-Mendoza A., Gonzalez-Aguilar G.A., Martinez-Tellez M.A., Nazzaro F., Ayala-Zavala J.F. (2019). Virulence of *Pseudomonas aeruginosa* exposed to carvacrol: Alterations of the Quorum sensing at enzymatic and gene levels. J. Cell Commun. Signal..

[16] Khan S.T., Khan M., Ahmad J., Wahab R., Abd-Elkader O.H., Musarrat J., Alkhathlan H.Z., Al-Kedhairy A.A. (2017). Thymol and carvacrol induce autolysis, stress, growth inhibition and reduce the biofilm formation by *Streptococcus mutans*. AMB Express.

[17] Koraichi Saad I., Hassan L., Ghizlane Z., Hind M., Adnane R. (2011). Carvacrol and thymol components inhibiting *Pseudomonas aeruginosa* adherence and biofilm formation. Afr. J. Microbiol. Res..

[18] Khan I., Bahuguna A., Kumar P., Bajpai V.K., Kang S.C. (2017). Antimicrobial Potential of Carvacrol against Uropathogenic *Escherichia coli* via Membrane Disruption, Depolarization, and Reactive Oxygen Species Generation. Front. Microbiol..

[19] Shinde P., Agraval H., Srivastav A.K., Yadav U.C., Kumar U. (2020). Physico-chemical characterization of carvacrol loaded zein nanoparticles for enhanced anticancer activity and investigation of molecular interactions between them by molecular docking. Int. J. Pharm..

[20] Ilk S., Sağlam N., Özgen M., Korkusuz F. (2017). Chitosan nanoparticles enhances the anti-quorum sensing activity of kaempferol. Int. J. Biol. Macromol..

[21] Liu Y., Shi L., Su L., van der Mei H.C., Jutte P.C., Ren Y., Busscher H.J. (2019). Nanotechnology-based antimicrobials and delivery systems for biofilm-infection control. Chem. Soc. Rev..

[22] Mu H., Tang J., Liu Q., Sun C., Wang T., Duan J. (2016). Potent antibacterial nanoparticles against biofilm and intracellular bacteria. Sci. Rep..

[23] Feyzioglu G.C., Tornuk F. (2016). Development of chitosan nanoparticles loaded with summer savory *(Satureja hortensis* L.) essential oil for antimicrobial and antioxidant delivery applications. LWT.

[24] Tran T.-T., Hadinoto K. (2021). A Potential Quorum-sensing inhibitor for bronchiectasis therapy: Quercetin–chitosan nanoparticle complex exhibiting superior inhibition of biofilm formation and swimming motility of *Pseudomonas aeruginosa* to the native quercetin. Int. J. Mol. Sci..

[25] Li X., Yeh Y.-C., Giri K., Mout R., Landis R.F., Prakash Y., Rotello V.M. (2015). Control of nanoparticle penetration into biofilms through surface design. Chem. Commun..

[26] Shebl R.I., Farouk F., Azzazy H.M.E.-S. (2017). Effect of surface charge and hydrophobicity modulation on the antibacterial and antibiofilm potential of magnetic iron nanoparticles. J. Nanomater..

[27] Philippova O., Korchagina E. (2012). Chitosan and its hydrophobic derivatives: Preparation and aggregation in dilute aqueous solutions. Polym. Sci. Ser. A.

[28] Zhu X., Su M., Tang S., Wang L., Liang X., Meng F., Hong Y., Xu Z. (2012). Synthesis of thiolated chitosan and preparation nanoparticles with sodium alginate for ocular drug delivery. Mol. Vis..

[29] Tapia-Rodriguez M.R., Hernandez-Mendoza A., Gonzalez-Aguilar G.A., Martinez-Tellez M.A., Martins C.M., Ayala-Zavala J.F. (2017). Carvacrol as potential quorum sensing inhibitor of *Pseudomonas aeruginosa* and biofilm production on stainless steel surfaces. Food Control.

[30] Qu L., She P., Wang Y., Liu F., Zhang D., Chen L., Luo Z., Xu H., Qi Y., Wu Y. (2016). Effects of norspermidine on *Pseudomonas aeruginosa* biofilm formation and eradication. Microbiologyopen.

[31] Nafee N., Husari A., Maurer C.K., Lu C., de Rossi C., Steinbach A., Hartmann R.W., Lehr C.-M., Schneider M. (2014). Antibiotic-free nanotherapeutics: Ultra-small, mucus-penetrating solid lipid nanoparticles enhance the pulmonary delivery and anti-virulence efficacy of novel quorum sensing inhibitors. J. Control Release.

[32] Tan Y., Ma S., Leonhard M., Moser D., Haselmann G.M., Wang J., Eder D., Schneider-Stickler B. (2018). Enhancing antibiofilm activity with functional chitosan nanoparticles targeting biofilm cells and biofilm matrix. Carbohydr. Polym..

[33] López-Oyama A.B., Taboada P., Burboa M.G., Rodríguez E., Mosquera V., Valdez M.A. (2011). Interaction of the cationic peptide bactenecin with mixed phospholipid monolayers at the air–water interface. J. Colloid Interface Sci..

[34] Freudenthal O. (2016). Study of the Action of Antimicrobial Peptides by Spectroscopic Methods: From Model Membrane to Bacterial Biofilm.

[35] Nowotarska S.W., Nowotarski K.J., Friedman M., Situ C. (2014). Effect of structure on the interactions between five natural antimicrobial compounds and phospholipids of bacterial cell membrane on model monolayers. Molecules.

[36] Bernal-Mercado A.T., Gutierrez-Pacheco M.M., Encinas-Basurto D., Mata-Haro V., Lopez-Zavala A.A., Islas-Osuna M.A., Gonzalez-Aguilar G.A., Ayala-Zavala J.F. (2020). Synergistic mode of action of catechin, vanillic and protocatechuic acids to inhibit the adhesion of uropathogenic *Escherichia coli* on silicone surfaces. J. Appl. Microbiol..

[37] Giri K., Yepes L.R., Duncan B., Parameswaran P.K., Yan B., Jiang Y., Bilska M., Moyano D.F., Thompson M.A., Rotello V.M. (2015). Targeting bacterial biofilms via surface engineering of gold nanoparticles. RSC Adv..

[38] Goodman C.M., McCusker C.D., Yilmaz T., Rotello V.M. (2004). Toxicity of gold nanoparticles functionalized with cationic and anionic side chains. Bioconjug. Chem..

[39] Gupta A., Landis R.F., Rotello V.M. (2016). Nanoparticle-based antimicrobials: Surface functionality is critical. F1000Res..

[40] Peulen T.-O., Wilkinson K. (2011). Diffusion of nanoparticles in a biofilm. Environ. Sci. Technol..

[41] Upadhyay A., Arsi K., Wagle B.R., Upadhyaya I., Shrestha S., Donoghue A.M., Donoghue D.J. (2017). Trans-cinnamaldehyde, carvacrol, and eugenol reduce *Campylobacter jejuni* colonization factors and expression of virulence genes in vitro. Front. Microbiol..

[42] Tan Y., Leonhard M., Moser D., Ma S., Schneider-Stickler B. (2016). Long-term antibiofilm activity of carboxymethyl chitosan on mixed biofilm on silicone. Laryngoscope.

[43] Kilmury S.L., Burrows L.L. (2018). The *Pseudomonas aeruginosa* PilSR two-component system regulates both twitching and swimming motilities. Mbio.

[44] Leja K., Drożdżyńska A., Majcher M., Kowalczewski P.Ł., Czaczyk K. (2019). Influence of sub-inhibitory concentration of selected plant essential oils on the physical and biochemical properties of *Pseudomonas orientalis*. Open Chem..

[45] Van Alphen L.B., Burt S.A., Veenendaal A.K., Bleumink-Pluym N.M., Van Putten J.P. (2012). The natural antimicrobial carvacrol inhibits *Campylobacter jejuni* motility and infection of epithelial cells. PLoS ONE.

[46] Mohammed H.B., Rayyif S.M.I., Curutiu C., Birca A.C., Oprea O.-C., Grumezescu A.M., Ditu L.-M., Gheorghe I., Chifiriuc M.C., Mihaescu G. (2021). Eugenol-functionalized magnetite nanoparticles modulate virulence and persistence in *Pseudomonas aeruginosa* clinical strains. Molecules.

[47] Bose S.K., Nirbhavane P., Batra M., Chhibber S., Harjai K. (2020). Nanolipoidal α-terpineol modulates quorum sensing regulated virulence and biofilm formation in Pseudomonas aeruginosa. Nanomedicine.

[48] Subhaswaraj P., Barik S., Macha C., Chiranjeevi P.V., Siddhardha B. (2018). Anti quorum sensing and anti biofilm efficacy of cinnamaldehyde encapsulated chitosan nanoparticles against *Pseudomonas aeruginosa* PAO1. LWT.

[49] Luna M., Beltran O., Encinas-Basurto D.A., Ballesteros-Monrreal M.G., Topete A., Hassan N., López-Mata M.A., Reyes-Márquez V., Valdez M.A., Juarez J. (2022). High antibacterial performance of hydrophobic chitosan-based nanoparticles loaded with carvacrol. Colloids Surf. B..

[50] Robles E., Juárez J., Burboa M.G., Gutiérrez L.E., Taboada P., Mosquera V., Valdez M.A. (2014). Properties of insulin–chitosan complexes obtained by an alkylation reaction on chitosan. J. Appl. Polym. Sci..

[51] Almada M., Burboa G., Robles E., Gutiérrez L., Valdés M., Juárez J. (2014). Interaction and cytotoxic effects of hydrophobized chitosan nanoparticles on MDA-MB-231, HeLa and Arpe-19 cell lines. Curr. Top. Med. Chem..

[52] Hajimehdipoor H., Shekarchi M., Khanavi M., Adib N., Amri M. (2010). A validated high performance liquid chromatography method for the analysis of thymol and carvacrol in *Thymus vulgaris* L. volatile oil. Pharmacogn. Mag..

[53] Alvarez M.V., Ortega-Ramirez L.A., Gutierrez-Pacheco M.M., Bernal-Mercado A.T., Rodriguez-Garcia I., Gonzalez-Aguilar G.A., Ponce A., Moreira M.d.R., Roura S.I., Ayala-Zavala J.F. (2014). Oregano essential oil-pectin edible films as anti-quorum sensing and food antimicrobial agents. Front. Microbiol..

[54] Bernal-Mercado A.T., Vazquez-Armenta F.J., Tapia-Rodriguez M.R., Islas-Osuna M.A., Mata-Haro V., Gonzalez-Aguilar G.A., Lopez-Zavala A.A., Ayala-Zavala J.F. (2018). Comparison of single and combined use of catechin, protocatechuic, and vanillic acids as antioxidant and antibacterial agents against uropathogenic *Escherichia coli* at planktonic and biofilm levels. Molecules.

[55] Wang H., Yu X., Su C., Shi Y., Zhao L. (2018). Chitosan nanoparticles triggered the induction of ROS-mediated cytoprotective autophagy in cancer cells. Artif. Cells Nanomed. Biotechnol..

[56] Reynolds F., O’loughlin T., Weissleder R., Josephson L. (2005). Method of determining nanoparticle core weight. Anal. Chem..

[57] Broniatowski M., Macho I.S., Dynarowicz-Łątka P. (2005). Study of perfluorooctyl-n-alkanes monolayers at the air–water interface. Thin Solid Film..

